# Predicting EQ-5D-3L utility values from clinical data in a prospective cohort of kidney transplant recipients

**DOI:** 10.1007/s10198-025-01802-6

**Published:** 2025-06-11

**Authors:** V. Bonnemains, Y. Foucher, P. Tessier, C. David, M. Giral, E. Dantan

**Affiliations:** 1 Nantes Université, Univ Tours, CHU Nantes, INSERM, MethodS in Patients-Centered Outcomes and HEalth Research SPHERE, 44000 Nantes, France; 2 INSERM, CIC-1402, Centre Hospitalier Universitaire de Poitiers, Université de Poitiers, Poitiers, France; 3 CRTI UMR 1064, Inserm, Université de Nantes, ITUN, CHU Nantes, RTRS Centaure, Nantes, France; 4 Centre d’Investigation Clinique en Biothérapie, Nantes, France

**Keywords:** Health-state utility, Mapping, EQ-5D, Kidney transplantation, Longitudinal prediction

## Abstract

**Objectives:**

Modelling health-state utility values (HSUVs) from clinical data offers a means to conduct retrospective cost-effectiveness analyses using clinical studies that did not collect direct HSUV measures. Such studies can support the efficient allocation of resources in kidney transplantation (KT). We aim to model KT recipients' EQ-5D-3L HSUVs using routinely collected clinical data.

**Methods:**

From a French observational multicentric prospective cohort, we included 2,787 adult recipients of a first or second single renal graft transplanted between January 2014 and December 2021 who completed 5,679 EQ-5D-3L questionnaires post-KT, from which the HSUVs were calculated. Considering two time periods before and after 1-year post-KT, we estimated a linear mixed effect model (LME), a mixed adjusted limited dependent variable mixture model, and beta and two-part beta mixed models. We compared their predictive performances in terms of precision and calibration.

**Results:**

In each model, recipient age, female sex, higher body mass index, presence of comorbidities and time spent on dialysis prior to KT were associated with lower HSUVs. The predicted HSUVs increased during the first year post-KT before slowly decreasing afterwards. The two-part beta mixed model resulted in the most precise predictions but showed poor calibration. The LME was associated with better calibration than the other models.

**Conclusions:**

Our study illustrates the importance of estimating longitudinal predictive algorithms to consider possible time variations in HSUVs. We provide an online calculator for predicting the HSUVs of KT recipients over time. Future studies in international cohorts are important to support the external validity of our results.

**Supplementary Information:**

The online version contains supplementary material available at 10.1007/s10198-025-01802-6.

## Introduction

When possible, kidney transplantation (KT) is the preferred renal replacement therapy for end-stage renal disease (ESRD) in terms of morbidity, mortality, and quality of life [1, 2]. Associated with lower costs compared to peritoneal dialysis and haemodialysis [3], KT has been reported to be cost-effective in several studies [4, 5]. Nevertheless, the assessment of solutions aimed at improving transplantation procedures and patients’ management could benefit from further cost-effectiveness studies.

Such studies often rely on modelling approaches such as Markov models to represent costs and Quality-Adjusted Life-Years (QALYs) over a long time horizon [6–8]. This requires that published health states utility values (HSUVs) be available to estimate QALYs. However, model-based cost-effectiveness studies cannot always rely on longitudinal HSUVs, which are rarely collected in practice. Thus, the possibility to predict the evolution over time of KT recipients’ HSUVs from routinely collected clinical data could be helpful to provide more accurate estimates of QALYs in model-based cost-effectiveness studies and in studies that did not collect preference-based measures of Health-Related Quality of Life (HRQoL).

Li et al. recently proposed models to predict 6-month post-transplantation EQ-5D HSUVs for KT recipients from clinical variables, arguing that HSUV at this time offered a stable reflection of post-KT HRQoL [9]. This assumption of a stable health state and corresponding utility value is questionable. Indeed, answers to the EQ-5D questionnaire are characterized by an individual variability along the KT recipient follow-up, which makes the corresponding HSUVs time-dependent. Additionally, Galichon et al. considered two clinical phases in the post-KT follow-up: one phase associated with post-operation complications and acute rejection episodes, usually considered as the first year post-transplantation, followed by a chronic phase [10]. They reported a higher but decreasing graft failure rate during the first year post-KT, followed by a steadier rate thereafter. To the best of our knowledge, only Li et al. [9] proposed a predictive model of HSUVs in KT. Given the endogenous nature of HSUVs and the two clinical phases described by Galichon et al. [10], it appears relevant to consider the HSUV of KT recipients as a time-dependent outcome.

Kontodimopoulos et al. reported that the estimation of a prediction model using only baseline data could lead to biased QALY predictions [11]. Gonçalves et al. further stressed the importance of including all time points in the analysis [12]. Although linear models have commonly been employed to model HSUVs in cross sectional studies [13], linear mixed models enable the consideration of intra-class correlations, for example, those induced by individual repeated measures in longitudinal data [14]. However, this relies on several assumptions with theoretical limitations in the context of HSUV modelling: the unboundedness, unimodality and symmetry of the normal distribution [15]. More recently, beta regression models have been proposed to consider the bounded and skewed nature of the HSUV distribution [16, 17], as well as mixture models to account for possible bimodality [18]. Nevertheless, their random effect extensions for longitudinal data remain sparsely studied and have never been investigated in the specific context of kidney transplantation.

Using questionnaires collected longitudinally since 2014 for a French cohort of KT patients, the present study aims to propose a new model to predict the post-KT evolution of EQ-5D HSUVs until they returned to dialysis or died from clinical information, as well as an online calculator allowing investigators to apply this model to their data. For this purpose, we compare the performances of several models to predict time-dependent HSUVs. This would provide new insights on the model family that best fits such data. This study also provides an external validation of Li et al.’s model.

## Materials and methods

### Study population

**Fig. 1:**
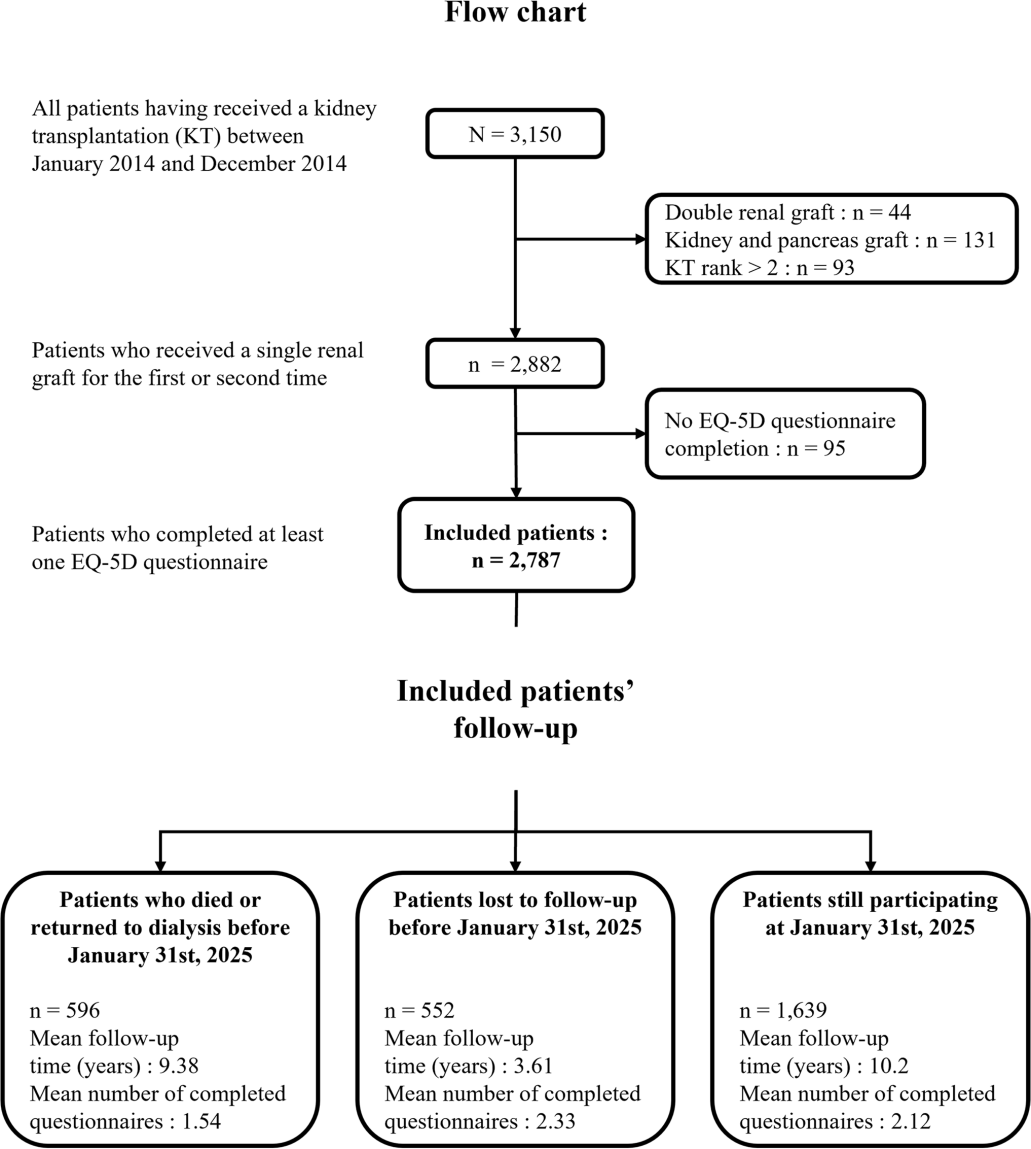
Flow chart of the cohort study design

Data were extracted from the French multicentric, observational and prospective DIVAT cohort (Données Informatisées et VAlidées en Transplantation, www.divat.fr, CNIL no. 914184, ClinicalTrials.gov recording NCT02900040). All participants gave informed consent. Since January 2014, patients have been encouraged to complete self-administered EQ-5D-3L questionnaires at each anniversary of the transplantation in 6 centres until they returned to dialysis or died, while being able to do so with no restrictions in the period of collection. We considered all the completed questionnaires regardless of the time of completion. The inclusion criteria for this study were: adult recipients of a first or second single renal graft and transplanted between January 2014 and December 2021. This led to 2,787 included patients (Fig. 1). Among them, 12 patients (0.4%) completed EQ-5D questionnaires during their first and second transplantations. This low number of occurrences did not allow the consideration of transplantation as a second grouping level in our analyses. Therefore, we chose to consider only the first transplantation for these 12 patients so that no patient was included more than once. There were no multiorgan transplant recipients in the study.

### Available data

HSUVs were obtained from the patients responses to the EQ-5D-3L questionnaire [19]. This questionnaire is made up of five questions, each exploring one dimension of the respondent’s quality of life: mobility, self-care, usual activities, pain/discomfort, and anxiety/depression. In the 3L version, each question can be given a 3-ordered-level answer (no problem, some problems and extreme problems) such that a higher level indicates more problem with the dimension under consideration. These questionnaires were converted into HSUVs using French published tariffs [20]. From expert background knowledge (M.G.) and considering the most frequent comorbidities occurring before kidney transplantation with ageing and chronic kidney insufficiency, the following baseline recipient characteristics were considered potential determinants of the HSUVs: age, sex, body mass index (BMI), history of comorbidities (diabetes, cardiovascular disease, neoplasia, hypertension), time spent on dialysis before transplantation, graft rank and cause of initial renal disease (recurrent nephropathy or not). Donor age was also included in the analysis.

### Statistical analyses

Recipient and donor characteristics were reported as the mean and standard deviation for continuous variables and as count and percentage for categorical variables. For the longitudinal analysis of HSUVs, we compared four multivariate mixed regression models: linear mixed model, mixed adjusted limited dependent variable mixture model (mixed ALDVMM) [18], mixed beta regression [16, 17] and mixed two-part beta regression [17] (details of each approach are provided in Online Resource 1).

For the linear mixed model and the mixed ALDVMM, we modelled the raw HSUVs. For the mixed beta and two-part beta regressions, we transformed the HSUVs $u$ so that they are restricted from 0 to 1 while the raw HSUVs resulting from the French EQ-5D-3L scoring system may range from −0.53 (${u}_{min}$) to 1.00 (${u}_{max}$): $y=({u}_{max}-u)/\left({u}_{max}-{u}_{min}\right)$, as suggested by Brazier et al. [21]. Note that in doing so, we thus modelled the rescaled decrement $y$ in patients’ HSUVs $u$ compared to perfect health. The two-part beta model corresponds to a zero-inflated beta-regression, possibly interesting to consider the mass of observations at the maximum value of 1, which correspond to a decrement $y$ of 0. We compressed the $y$-values into ${y}^{*}$ so that they never reach the boundary values using the method suggested by Hunger et al. [16], i.e., ${y}^{*}=\left[y\times \left(N-1\right)+0.5)\right]/N$ where $N$ denotes the total number of observations. We modelled the corresponding longitudinal decrements using the $logit$ link function. The beta-based model predictions were then back-transformed to obtain HSUV predictions ranging from −0.53 to 1, comparable to those of the other models. Hence, predicted HSUVs are the outcome of each model, regardless of whether it belongs to a linear-based or beta-based family.

For each model, we first included the time effect considering two evolution slopes before and after 1-year post-KT [10]. Then, all the characteristics listed in Table 1 were considered possible baseline predictor and selected following the same procedure on the basis of likelihood-ratio tests: 1) univariate selection (p < 0.20), 2) and then a forward selection (p < 0.05). ALDVMMs including 1 and 2 classes were estimated using this procedure and the one with the lowest BIC was ultimately retained.

**Table 1:** Characteristics of the recipients and donors in the cohort at the time of transplantation (*n* = 2,787)

Quantitative characteristics	Missing	Mean (± SD)
Recipient age (years)	0	50.0 (± 14.2)
Donor age (years)	7	51.6 (± 15.7)
Time spent on dialysis (years)	12	2.5 (± 3.3)
**Categorical characteristics**	**Missing**	**Effective (%)**
Recipient men	0	1,757 (63.0)
Recipient Body mass index	2	
Less than 18 kg/m^2^ In between 18 and 30 kg/m^2^ Higher than 30 kg/m^2^		119 (4.3) 2,331 (83.7) 335 (12.0)
First transplantation	0	2,378 (85.3)
Recipient history of diabetes	0	378 (13.6)
Recipient history of cardiovascular disease	0	961 (34.5)
Recipient history of neoplasia	0	320 (11.5)
Recipient history of hypertension	0	2,414 (86.6)
Relapsing initial nephropathy	0	784 (28.1)

Missing, number of missing values; SD, standard deviation

We assessed the goodness of fit as follows. Performances were reported with root mean squared error (RMSE) and mean absolute error (MAE). We also evaluated the models’ calibration, i.e., their ability to accurately predict HSUVs across the whole prediction range. The models’ calibrations were graphically evaluated by comparing the observed versus predicted HSUVs in subgroups corresponding to each decile of predictions [22]. The calibration curve was obtained by linearly regressing the observed HSUVs and the predicted deciles [23]. The closer the regression coefficients of this linear regression are to an intercept of 0 and a slope of 1, the more the calibration can be considered reasonable. We also evaluated the model proposed by Li et al. using the same metrics. We performed a sensitivity analysis by fitting the models only on first-time KT recipients (Online Resource 2).

All analyses were performed using R software version 4.3.1 [24]. We provide the complete R code enabling HSUVs prediction under a gitlab repository, as well as a user-friendly R Shiny application available at https://sphere-inserm.fr/en/jm-qalys.

## Results

### Description of baseline characteristics

The demographic and clinical characteristics of the KT recipients are reported in Table 1. Among the 2,789 patients, the mean age at transplantation was 50.0 (± 14.2) years and the mean BMI was 24.6 (± 4.4) kg/m^2^. The studied sample included 1,757 men (63.0%), 378 patients (13.6%) with history of diabetes, 961 (34.5%) with history of cardiovascular disease and 2,378 (85.3%) transplanted for the first time.

### Description of the follow-up

**Fig. 2:**
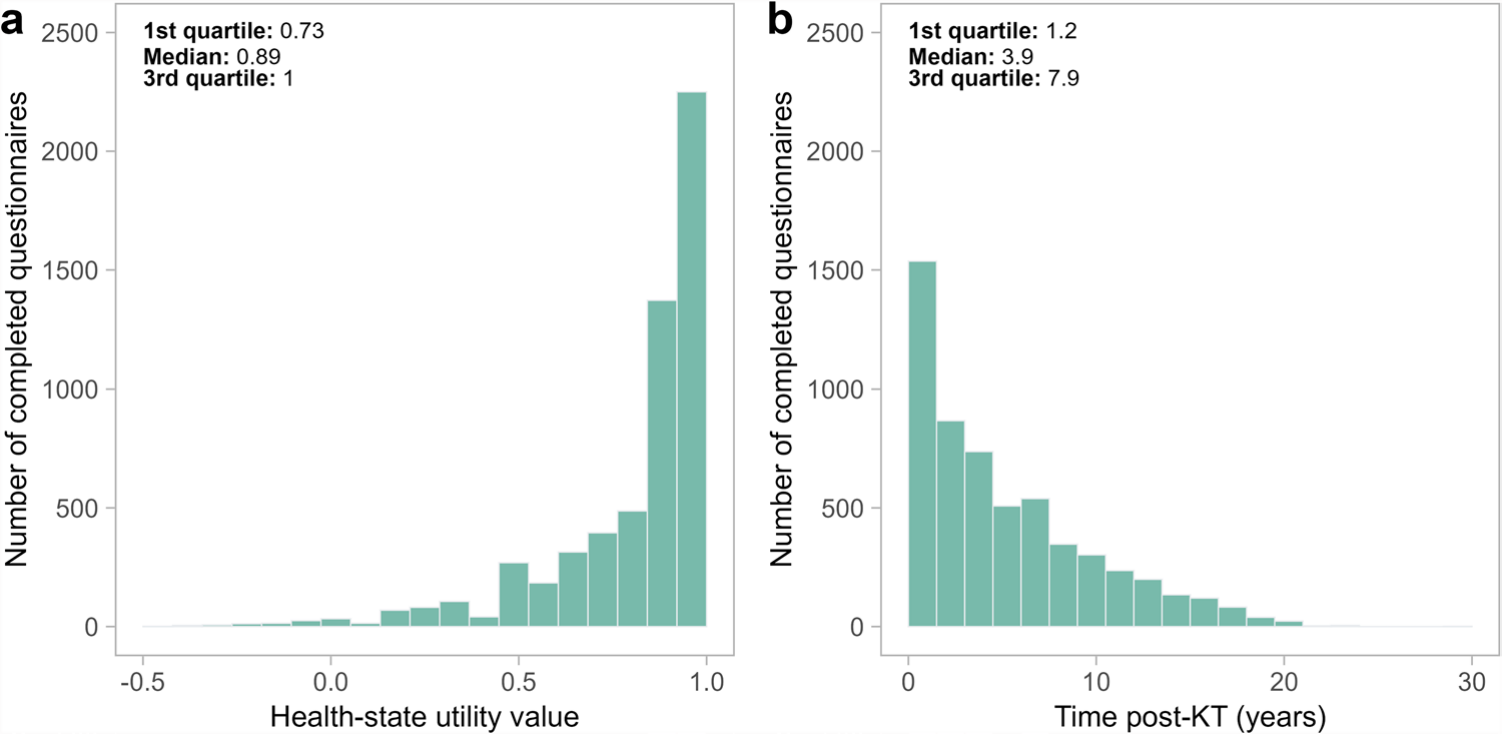
Distributions for EQ-5D-3L health-state utility values (part a) and EQ-5D-3L questionnaire completion time post-kidney transplantation (KT) (part b)

In the whole studied sample, 5,679 completed EQ-5D-3L questionnaires were collected. The number of questionnaires per patient ranged from 1 to 10: 1,430 (51.3%) patients completed one questionnaire, 634 (22.7%) completed two and 723 (25.9%) completed at least three. HSUVs and completion time distributions are represented in Figs. 2a and b.

Among the 5,679 completed questionnaires, 1,132 (19.9%) were completed during the first year post-KT, while 4,547 (80.1%) were completed later, up to 29.5 years post-KT. Patients’ median latest questionnaire completion time was 5.0 years. The EQ-5D HSUVs ranged between −0.497 and 1.00 throughout the questionnaires, with a mean of 0.82 (± 0.23). Few patients reported low HSUVs (first decile = 0.487). As previously described [18], many patients (*n* = 2,250, 39.6%) reported the best possible health-state, which corresponds to a HSUV of 1. Online Resource 3 illustrates the longitudinal evolution of the observed HSUVs in four subgroups of patients according to sex and diabetes (Figure S1).

### Selection of the models

**Fig. 3:**
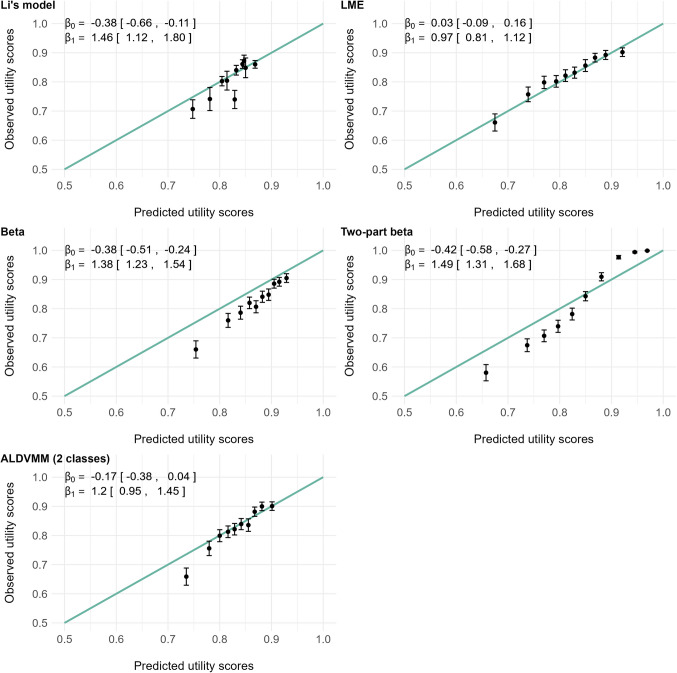
Predicted versus observed health-state utility values (HSUVs) for the five models. The predicted HSUVs were grouped by deciles, and the black dots represent the means of the observed and predicted HSUVs for each group. The green lines represent perfect calibrations. ${\beta }_{0}$ and ${\beta }_{1}$ are the intercept and slope, respectively, obtained by linearly regressing* the observed HSUVs and the ten predicted deciles (a perfect calibration corresponding to ${\beta }_{0}=0$ and ${\beta }_{1}=1$). LME: linear mixed effect model; ALDVMM: adjusted limited dependent variable mixture model. * Li’s model’s 5th decile and the 2-class ALDVMM’s 1st decile were detected as outliers by analysing studentized residuals and Cook’s distance and excluded for ${\beta }_{0}$ and ${\beta }_{1}$ estimation

Calibration plots (Fig. 3) revealed a good adequation between predicted and observed HSUVs for the linear mixed model, which outperformed the other models in this area.

The 2-class ALDVMM, which resulted in a lower BIC than the 1-class alternative, was selected as the candidate ALDVMM. It showed a good calibration across most of the prediction range, except the lowest decile which was overestimated. The beta and 2-part beta models both showed a poor calibration across the whole prediction range: the first consistently overestimated HSUVs, while the second overestimated low scores and underestimated high ones. The model proposed by Li et al. also substantially overestimated the HSUVs in our sample.

The RMSE, MAE and prediction range resulting of each model’s predictions are reported in Table 2. The inclusion of a larger set of covariates along with the consideration of questionnaires completed at different times of patients’ post-KT follow-up resulted in quite larger prediction ranges than those of the model proposed by Li et al. This was particularly the case for the two-part beta mixed model, which was able to predict both lower and higher HSUVs than the other models, and the closest HSUV prediction to 1 (prediction range from 0.357 to 0.990). This is further illustrated by Online Resource 3, in which the longitudinal predictions resulting from each model in four subgroups of patients according to sex and diabetes were represented along with the time spent since KT (Figure S2). Despite its poor calibration, the two-part beta model outperformed the other models both in terms of RMSE and MAE. Apart from this model, very little differences appeared from one model to another in terms of prediction errors.

**Table 2:** Root mean squared error (RMSE), mean absolute error (MAE) and prediction range for each model

	RMSE	MAE	Prediction range
Linear mixed model	0.223	0.165	[0.448, 0.975]
Beta mixed model	0.228	0.154	[0.415, 0.952]
Two-part beta mixed model	0.192	0.127	[0.357, 0.990]
2-class mixed ALDVMM	0.225	0.162	[0.625, 0.932]
Model proposed by Li et al.^*^	0.230	0.169	[0.718, 0.883]

^*^Model 7 from Li et al.’s Table 3, including recipients’ age, diabetic status and sex as predictors

### The linear mixed model for HSUV prediction

The linear mixed model, which showed the best calibration, is presented in Table 3. Being a woman, recipient age, history of diabetes, history of cardiovascular disease, a BMI greater than 30 kg/m^2^, and time in dialysis, were associated with lower baseline HSUVs. The most significant characteristics were diabetic status (−0.091 ± 0.013) and female sex (−0.074 ± 0.009), whereas recipient age (−0.002 ± 0.0003), time spent on dialysis (−0.009 ± 0.001), having a BMI greater than 30 kg/m^2^ (−0.037 ± 0.013) or the presence of cardiovascular comorbidities (0.036 ± 0.009) resulted in significant but lower decreases in HSUVs. Moreover, the HSUV increased during the first year (slope of the evolution (years) = + 0.059 ± 0.012), whereas it decreased afterwards (slope of the evolution (years) = −0.006 ± 0.001), suggesting a rapid increase of HSUV during the first year post-KT followed by a slow decrease thereafter.

**Table 3:** Multivariate linear mixed model of patients’ health-state utility values (*n* = 2,774, 13 patients excluded due to missing data on adjustment covariates)

	Coefficients	95% CI	p
**Baseline predictors**			
Age (years)	−0.002	[−0.003, −0.001]	< 0.001
Sex (men versus women)	0.074	[0.057, 0.091]	< 0.001
Body mass index (> 30 kg/m^2^, vs ≤ 30)	−0.037	[−0.063, −0.012]	0.004
History of diabetes (yes versus no)	−0.091	[−0.116, −0.066]	< 0.001
History of cardiovascular disease (yes versus no)	−0.036	[−0.054, −0.018]	< 0.001
Time spent on dialysis (years)	−0.009	[−0.011, −0.006]	< 0.001
**Slopes of the evolution (years)**			
Before 1-year post-transplantation	0.059	[0.035, 0.083]	< 0.001
After 1-year post-transplantation	−0.006	[−0.007, −0.004]	< 0.001

Intercept: 0.881 (95% CI [0.842, 0.921]), SD of the random intercept: 0.231 (95% CI [0.211, 0.252]), SD of the random slope in the first year post-KT: 0.213 (95% CI [0.186, 0.241]), SD of the residuals: 0.122 (95% CI [0.118, 0.125])

*CI*, confidence interval; *KT*, kidney transplantation; *SD*, standard deviation

### The two-part beta mixed model for HSUV prediction

The two-part beta mixed model, which showed the best precision, is presented in Table 4. Consistent with the linear mixed model outcome, being a woman, recipient age, history of diabetes, history of cardiovascular disease, having a BMI greater than 30 kg/m^2^ and time in dialysis, were associated with both lower probability of reporting the maximum HSUV at baseline and higher decrements from it, i.e., lower baseline HSUVs.

**Table 4:** Multivariate two-part beta mixed model of patients rescaled decrement in health-state utility values (HSUVs) (*n* = 2,774, 13 patients excluded due to missing data on adjustment covariates). A greater decrement indicates a lower HSUV

*Zero-inflation model*	Coef	95% CI	p
**Baseline predictors**			
Age (years)	−0.025	[−0.035, −0.016]	< 0.001
Sex (men vs. women)	1.055	[0.799, 1.310]	< 0.001
Body mass index (> 30 kg/m^2^, vs ≤ 30)	−0.464	[−0.837, −0.090]	0.015
Recipient history of diabetes (yes vs. no)	−0.534	[−0.904, −0.164]	0.005
Recipient history of cardiovascular disease (yes vs. no)	−0.541	[−0.807, −0.276]	< 0.001
Time spent on dialysis (years)	−0.079	[−0.117, −0.041]	< 0.001
**Slope of the evolution (years)**			
≤ 1-year post-KT	0.993	[0.631, 1.355]	< 0.001
> 1-year post-KT	−0.070	[−0.096, −0.044]	< 0.001
Intercept: −0.403 (95% CI [−0.982, 0.176]), SD of the random intercept: 2.112 (95% CI [1.895, 2.353])			
***Conditional model***	**Coef**	**95% CI**	**p**
***Baseline predictors***			
Age (years)	0.008	[0.005, 0.010]	< 0.001
Sex (men vs. women)	−0.229	[−0.304, −0.153]	< 0.001
Body mass index (> 30 kg/m^2^, vs ≤ 30)	0.120	[0.012, 0.230]	0.030
Recipient history of diabetes (yes vs. no)	0.382	[0.276, 0.488]	< 0.001
Recipient history of cardiovascular disease (yes vs. no)	0.107	[0.026, 0.188]	0.009
Time spent on dialysis (years)	0.030	[0.019, 0.040]	< 0.001
**Slope of the evolution (years)**			
≤ 1-year post-KT	−0.222	[−0.340, −0.105]	< 0.001
> 1-year post-KT	0.022	[0.014, 0.030]	< 0.001

Intercept: −1.919 (95% CI [−2.104, −1.734]), SD of the random intercept: 0.766 (95% CI [0.676, 0.868]), SD of the random slope in the first year post-KT: 0.872 (95% CI [0.740, 1.027])

*CI*, confidence interval; *KT*, kidney transplantation; *SD*, standard deviation

Diabetic status and female sex were again the most significant characteristics on the conditional model (respectfully, 0.382 ± 0.054 and 0.229 ± 0.039), while female sex and recipient age were the most significant characteristics on the zero-inflation model (respectfully, 1.055 ± 0.130 and −0.025 ± 0.005).

Time since KT was also estimated to have consistent effects with the linear mixed model, i.e., an increase in HSUVs and the probability of reporting the maximum HSUV during the first year post-KT (slope of the evolution (years) = −0.222 ± 0.060 in the conditional model and 0.993 ± 0.185 in the zero-inflation model) followed by a slow decrease thereafter (slope of the evolution (years) 0.022 ± 0.004 in the conditional model and −0.070 ± 0.013 in the zero-inflation model). The other candidate models are presented in Online Resource 1.

## Discussion

We found that the linear mixed model was overall better calibrated than the alternative models in predicting the post-KT evolution of patients’ EQ-5D-3L HSUVs until they returned to dialysis or died, although the two-part beta mixed model resulted in the most precise predictions. Carefully chosen, both models can be useful to predict HSUVs for future cost-effectiveness studies, especially because the model proposed by Li et al. was associated with an unsatisfactory calibration and less precise predictions.

Building regression models to predict HSUVs is challenging because their distributions typically exhibit particular features such as boundedness, right skewness, multimodality and a mass of observations at the maximum value [18]. The linear framework has been widely used but theoretically fails to account for these features, raising questions about its appropriateness [15]. ALDVMMs were developed to better account for HSUV properties [18, 25–29]. Beta regression provides another flexible framework able to handle bounded and skewed distributions [16, 17, 30]. In addition, modelling the longitudinal evolution of HSUVs requires the hierarchical structure of the data to be considered [12]. This can be done by considering random effects in the previous parametric regressions.

In our study, the linear mixed model resulted in predictions of HSUVs as precise as most concurrent models, despite the latter’s theoretically sounder properties. This is consistent with the existing literature, which often reports small differences between the different classes of models precision-wise, in both cross-sectional [17, 27–34] and longitudinal settings[26, 35–37]. The mixed 2-class ALDVMM predictions were well calibrated, except for the lowest HSUVs, which were overestimated. Although ALDVMMs have been reported to perform better, especially for low HSUVs [26, 37, 38], the linear mixed model showed good calibration in our application even for the lowest prediction decile. The KT context might explain this finding: in our data, the HSUV distribution was unimodal with only few extremely low HSUVs. Moreover, the linear mixed model predictions all lied inside the possible range of HSUVs, which cancels out the theoretical limitation due to their bounded nature. Our study also highlights the importance of providing disease-specific prediction algorithms, as no one-fits-all model seem to exist.

Both beta-based models showed calibration issues. These issues have been previously reported to varying degrees for both beta models [16, 17] and two-part beta models [39], despite some literature indicating better results for these types of models [30, 31]. Although the beta distribution seemed a promising framework due to its boundedness and the flexibility allowed by its two shape parameters, the assumption that HSUVs would be beta-distributed does not seem to fit our data. However, the addition of zero-inflation substantially enhanced the precision of the predictions, resulting in better RMSE and MAE than the concurrent models.

The discrepancy between the two-part beta mixed model’s poor calibration but good precision can be explained by the lower variance of its predictions, especially for high HSUVs, as shown in Fig. 3. Hence, the choice of whether using it or the linear mixed model comes down to a trade-off between bias and variance. While one can be tempted to retain the model with the lowest RMSE, which is a criterion that reflects both features, we rather advise using the linear mixed model. Indeed, the two-part beta mixed model’s overestimation of low HSUVs and underestimation of high ones would result in biased QALYs estimation, should the model be applied to populations at either end of the scale. Nevertheless, we propose the use of both models in an online calculator enabling HSUV predictions from KT recipients'clinical characteristics, available at https://sphere-inserm.fr/en/jm-qalys.

Several previous studies aimed to predict the post-KT HRQoL [9, 40–43]. However, most of them used non-preference-based HRQoL measures that are unsuitable to obtain HSUVs, which are necessary to estimate QALYs and perform cost-effectiveness analyses. To the best of our knowledge, Li et al. [9] is the only study that proposed such a predictive model. However, we reported a poor calibration from our French cohort. Moreover, it consists of time-fixed predictions, assuming that the 6-month post-KT HSUV adequately reflects the whole prediction time range, with a limited number of predictors. Despite these differences, our proposed models and Li et al.’s find consistent effects, i.e., a negative effect of age, diabetes and female sex on patients’ HSUVs. Our proposed models are also consistent with studies that reported the association between KT recipients’ clinical characteristics and non-preference-based HRQoL metrics [40–42]. Indeed, patient’s age, female sex, presence of comorbidities and time since dialysis were found to be associated with lower levels of various HRQoL dimensions. It is harder to compare the effect of time post-KT evaluated in our models to this literature, which reports different time effects depending on the HRQoL dimension, while not using the same modelling as us, i.e., two slopes before and after 1-year post-KT. However, our models’ estimates showing a substantial increase in HSUV during the first year post-KT followed by a steadier state thereafter matches the shape of graft failure rate over time reported by Galichon et al. [10], which arguably supports the clinical relevance of this modelling choice. The observed HSUVs in the DIVAT cohort were also consistent with the previously published results, although they concern non-French populations. Indeed, we reported a mean HSUV of 0.823 whereas Li et al. reported an average 6-month post-KT HSUV of 0.827, Liem et al. reported an average EQ-5D-3L HSUV of 0.81 and Wyld et al. reported an average HSUV of 0.82 [9, 44, 45].

Our study proposes models for the time-dependent prediction of HSUVs, which may be used in practice to address the limited availability of preference-based quality-of-life measures for KT patients. These models could help estimate QALYs [46] and facilitate retrospective cost-effectiveness analyses based on clinical trials, as well as quasiexperimental studies using observational datasets and cohorts that did not collect HSUVs. Predicting patients’ utility may also be useful for populating Markov models in economic evaluations. Model-based CEAs are frequently used in studies supporting reimbursement or pricing decisions, as seen in the UK and France, for instance, and inputs for these models may sometimes be difficult to obtain. Finally, predicted HSUVs could also be useful in developing tools that support clinical decision-making.

Our study suffers from several limitations. First, we encountered difficulties in estimating random slopes, 1,430 out of 2,787 (51.3%) patients having only one measure of HSUV. Updating the proposed models in a few years with a higher number of EQ-5D questionnaires per patient would be relevant, notably to increase the precision of the long-term evolution. Second, we could not evaluate the models’ performances using an external validation sample, which questions the generalization of our findings to other countries. Third, the HSUVs were measured with the EQ-5D-3L rather than the EQ-5D-5L, which results in a lower sensitivity and potential ceiling effects due to the lower number of distinguishable health states [47]. However, the EQ-5D-5L was not associated with a French value set before 2020 [48], hence the use of the three-level version in the DIVAT cohort. Fourth, we did not investigate every aspect of the ALDVMM framework. Notably, we did not consider the inclusion of covariates in the class membership probability, nor the existence of more than two latent classes in the population. We also did not consider beta-regression mixtures in our candidate models. Although its relative simplicity may explain the rather disappointing results of our mixed ALDVMM, fitting the latter was already quite a challenge, which dissuaded us to add further complexity. Investigating the relationship between disease-specific QoL measures and the EQ-5D-3L HSUVs in patients suffering from chronic kidney disease-associated pruritus, Hernandez Alava et al. reported the performance of two-class and three-class ALDVMMs [49]. They observed similar performances of the two models in terms of RMSE and MAE. Studying the existence of more than two latent classes in the mixed ALDVMM framework would be an interesting perspective for our work. We limited our results to two-class ALDVMM because of convergence issues noted for three-class ALDVMM. This may be due to the insufficient sample size and number of observations per patient with respect to model complexity. Moreover, studies having considered beta mixtures as an attempt to improve ALDVMMs did not report clear improvements [26–29]. Fifth, we considered the evolution of the HSUV in two time periods: before and after 1-year post-KT. Alternative time cut-offs could also be relevant. Additionally, the HSUV evolution could be decomposed into more than two phases. Considering two phases in the first year post-KT may be a modelling option since it could correspond to an acute risk of surgical complications in the very early posttransplantation times and a higher risk of acute graft rejection just after this initial phase. Distinguishing KT recipients’ medium-term and long-term HSUV evolution (e.g., from 1 to 10 years post-KT and after 10 years post-KT) could also be relevant, although it would require sufficient long-term follow-up. However, the considered time periods were consistent with both the graphical examination of the observed data and the clinical considerations reported by Galichon et al. [10]. Finally, we considered only baseline characteristics along with time since KT in our models. Considering post-KT variables such as graft function or the occurrence of events is an interesting perspective that may improve the calibration of HSUV predictions.

In conclusion, we propose two predictive models of the post-KT EQ-5D-3L HSUVs until they returned to dialysis or died based on routinely collected clinical data, and we report their goodness of fit compared to other regression methods. Both of them outperformed the model proposed by Li et al. [9] which was associated with a lack of calibration in our French cohort. Although we advertise for the use of the linear mixed model, both may constitute a useful tool to allow for future model-based cost-effectiveness studies or studies using information from clinical trials that did not directly collect HSUVs. Therefore, we provide an online application to predict KT recipients’ HSUVs using either model. Future studies applied to international cohorts are important to support the external validity of our results.

## Supplementary Information

Below is the link to the electronic supplementary material.

Supplementary file1 (PDF 157 KB)

Supplementary file2 (PDF 379 KB)

Supplementary file3 (PDF 561 KB)

## Data Availability

The data underlying this article could be shared on reasonable request to the corresponding author.

## Abbreviations

95% CI: 95% Confidence Interval
ALDVMM: Adjusted Limited Dependent Variable Mixture Model
BIC: Bayesian Information Criterion
BMI: Body Mass Index
DIVAT: Données Informatisées et VAlidées en Transplantation
ESRD: End-Stage Renal Disease
HRQoL: Health-Related Quality of Life
HSUV: Health State Utility Values
KT: Kidney Transplantation
LME: Linear Mixed Effect Model
MAE: Mean Absolute Error
NA: Not Available (missing data)
QALYs: Quality-Adjusted Life-Years
RMSE: Root Mean Squared Error
SD: Standard Deviation

## References

[1] Chaudhry, D., Chaudhry, A., Peracha, J., Sharif, A.: Survival for waitlisted kidney failure patients receiving transplantation versus remaining on waiting list: systematic review and meta-analysis. BMJ **376**, e068769 (2022). 10.1136/bmj-2021-068769 35232772 10.1136/bmj-2021-068769 PMC8886447

[2] Tonelli, M., Wiebe, N., Knoll, G., Bello, A., Browne, S., Jadhav, D., Klarenbach, S., Gill, J.: Systematic review: kidney transplantation compared with dialysis in clinically relevant outcomes. Am. J. Transplant. Off. J. Am. Soc. Transplant. Am. Soc. Transpl. Surg. **11**, 2093–2109 (2011). 10.1111/j.1600-6143.2011.03686.x 10.1111/j.1600-6143.2011.03686.x 21883901

[3] Zhang, Y., Gerdtham, U.-G., Rydell, H., Lundgren, T., Jarl, J.: Healthcare costs after kidney transplantation compared to dialysis based on propensity score methods and real world longitudinal register data from Sweden. Sci. Rep. **13**, 10730 (2023). 10.1038/s41598-023-37814-6 37400547 10.1038/s41598-023-37814-6 PMC10318027

[4] Yang, F., Liao, M., Wang, P., Yang, Z., Liu, Y.: The Cost-Effectiveness of Kidney Replacement Therapy Modalities: A Systematic Review of Full Economic Evaluations. Appl. Health Econ. Health Policy **19**, 163–180 (2021). 10.1007/s40258-020-00614-4 33047212 10.1007/s40258-020-00614-4 PMC7902583

[5] Winkelmayer, W.C., Weinstein, M.C., Mittleman, M.A., Glynn, R.J., Pliskin, J.S.: Health economic evaluations: the special case of end-stage renal disease treatment. Med. Decis. Mak. Int. J. Soc. Med. Decis. Mak. **22**, 417–430 (2002). 10.1177/027298902236927 10.1177/027298902236927 12365484

[6] Redeker, S., Ismail, S., Eeren, H.V., Massey, E.K., Weimar, W., Oppe, M., Busschbach, J., Kidney Team at Home consortium: A dynamic Markov model to assess the cost-effectiveness of the Kidney Team at Home intervention in The Netherlands. Eur. J. Health Econ HEPAC Health Econ. Prev. Care. **23**, 597–606 (2022). 10.1007/s10198-021-01383-0 10.1007/s10198-021-01383-0 PMC8513543 34647158

[7] Varnell, C.D., Rich, K.L., Modi, A.C., Hooper, D.K., Eckman, M.H.: A Cost-effectiveness analysis of adherence promotion strategies to improve rejection rates in adolescent kidney transplant recipients. Am. J. Kidney Dis Off. J. Natl. Kidney Found. **80**, 330–340 (2022). 10.1053/j.ajkd.2021.12.013 10.1053/j.ajkd.2021.12.013 PMC9398956 35227823

[8] Yang, F., Liao, M., Wang, P., Liu, Y.: Cost-effectiveness analysis of renal replacement therapy strategies in Guangzhou city, southern China. BMJ Open **11**, e039653 (2021). 10.1136/bmjopen-2020-039653 33550227 10.1136/bmjopen-2020-039653 PMC7925861

[9] Li, B., Cairns, J.A., Draper, H., Dudley, C., Forsythe, J.L., Johnson, R.J., Metcalfe, W., Oniscu, G.C., Ravanan, R., Robb, M.L., Roderick, P., Tomson, C.R., Watson, C.J.E., Bradley, J.A.: Estimating health-state utility values in kidney transplant recipients and waiting-list patients using the EQ-5D-5L. Value Health J. Int. Soc. Pharmacoeconomics Outcomes Res. **20**, 976–984 (2017). 10.1016/j.jval.2017.01.011 10.1016/j.jval.2017.01.011 PMC5541449 28712628

[10] Galichon, P., Xu-Dubois, Y.-C., Finianos, S., Hertig, A., Rondeau, E.: Clinical and histological predictors of long-term kidney graft survival. Nephrol. Dial. Transplant. Off. Publ. Eur. Dial Transpl. Assoc. - Eur. Ren. Assoc. **28**, 1362–1370 (2013) 10.1093/ndt/gfs606 23348884

[11] Kontodimopoulos, N., Bozios, P., Yfantopoulos, J., Niakas, D.: Longitudinal predictive ability of mapping models: examining post-intervention EQ-5D utilities derived from baseline MHAQ data in rheumatoid arthritis patients. Eur. J. Health Econ HEPAC Health Econ. Prev. Care. **14**, 307–314 (2013). 10.1007/s10198-012-0376-9 10.1007/s10198-012-0376-9 22252308

[12] Oliveira Gonçalves, A.S., Werdin, S., Kurth, T., Panteli, D.: Mapping studies to estimate health-state utilities from nonpreference-based outcome measures: A systematic review on how repeated measurements are taken into account. Value Health. **26**, 589–597 (2023). 10.1016/j.jval.2022.09.2477 36371289 10.1016/j.jval.2022.09.2477

[13] Brazier, J.E., Yang, Y., Tsuchiya, A., Rowen, D.L.: A review of studies mapping (or cross walking) non-preference based measures of health to generic preference-based measures. Eur. J. Health Econ. **11**, 215–225 (2010). 10.1007/s10198-009-0168-z 19585162 10.1007/s10198-009-0168-z

[14] Laird, N.M., Ware, J.H.: Random-Effects Models for Longitudinal Data. Biometrics **38**, 963–974 (1982). 10.2307/2529876 7168798

[15] Wailoo, A.J., Hernandez-Alava, M., Manca, A., Mejia, A., Ray, J., Crawford, B., Botteman, M., Busschbach, J.: Mapping to Estimate Health-State Utility from Non-Preference-Based Outcome Measures: An ISPOR Good Practices for Outcomes Research Task Force Report. Value Health J. Int. Soc. Pharmacoeconomics Outcomes Res. **20**, 18–27 (2017). 10.1016/j.jval.2016.11.006 10.1016/j.jval.2016.11.006 28212961

[16] Hunger, M., Döring, A., Holle, R.: Longitudinal beta regression models for analyzing health-related quality of life scores over time. BMC Med. Res. Methodol. **12**, 144 (2012). 10.1186/1471-2288-12-144 22984825 10.1186/1471-2288-12-144 PMC3528618

[17] Basu, A., Manca, A.: Regression estimators for generic health-related quality of life and quality-adjusted life years. Med. Decis. Mak. Int. J. Soc. Med. Decis. Mak. **32**, 56–69 (2012). 10.1177/0272989X11416988 10.1177/0272989X11416988 PMC4575808 22009667

[18] Hernández Alava, M., Wailoo, A.J., Ara, R.: Tails from the peak district: adjusted limited dependent variable mixture models of EQ-5D questionnaire health state utility values. Value Health J. Int. Soc. Pharmacoeconomics Outcomes Res. **15**, 550–561 (2012). 10.1016/j.jval.2011.12.014 10.1016/j.jval.2011.12.014 22583466

[19] Rabin, R., de Charro, F.: EQ-5D: a measure of health status from the EuroQol Group. Ann. Med. **33**, 337–343 (2001). 10.3109/07853890109002087 11491192 10.3109/07853890109002087

[20] Chevalier, J., de Pouvourville, G.: Valuing EQ-5D using time trade-off in France. Eur. J. Health Econ HEPAC Health Econ. Prev. Care. **14**, 57–66 (2013). 10.1007/s10198-011-0351-x 10.1007/s10198-011-0351-x 21935715

[21] Brazier, J., Ratcliffe, J., Salomon, J., Tsuchiya, A.: Measuring and Valuing Health Benefits for Economic Evaluation. Oxford University Press (2016)

[22] Steyerberg, E.W., Vickers, A.J., Cook, N.R., Gerds, T., Gonen, M., Obuchowski, N., Pencina, M.J., Kattan, M.W.: Assessing the performance of prediction models: a framework for traditional and novel measures. Epidemiol. Camb. Mass. **21**, 128–138 (2010). 10.1097/EDE.0b013e3181c30fb2 10.1097/EDE.0b013e3181c30fb2 PMC3575184 20010215

[23] Crowson, C.S., Atkinson, E.J., Therneau, T.M.: Assessing calibration of prognostic risk scores. Stat. Methods Med. Res. 1–15 (2013) 10.1177/0962280213497434 PMC3933449 23907781

[24] R Development Core Team, .: R: A Language and Environment for Statistical Computing. , Vienna, Austria (2010)

[25] Pennington, B.M., Hernández-Alava, M., Hykin, P., Sivaprasad, S., Flight, L., Alshreef, A., Brazier, J.: Mapping From Visual Acuity to EQ-5D, EQ-5D With Vision Bolt-On, and VFQ-UI in Patients With Macular Edema in the LEAVO Trial. Value Health J. Int. Soc. Pharmacoeconomics Outcomes Res. **23**, 928–935 (2020). 10.1016/j.jval.2020.03.008 10.1016/j.jval.2020.03.008 PMC7427317 32762995

[26] Hernández Alava, M., Wailoo, A., Pudney, S., Gray, L., Manca, A.: Mapping clinical outcomes to generic preference-based outcome measures: development and comparison of methods. Health Technol. Assess. Winch. Engl. **24**, 1–68 (2020). 10.3310/hta24340 10.3310/hta24340 PMC7357250 32613941

[27] Aghdaee, M., Gu, Y., Sinha, K., Parkinson, B., Sharma, R., Cutler, H.: Mapping the patient-reported outcomes measurement information system (PROMIS-29) to EQ-5D-5L. Pharmacoeconomics **41**, 187–198 (2023). 10.1007/s40273-022-01157-3 36336773 10.1007/s40273-022-01157-3 PMC9883346

[28] Gray, L.A., Hernández Alava, M., Wailoo, A.J.: Development of Methods for the Mapping of Utilities Using Mixture Models: Mapping the AQLQ-S to the EQ-5D-5L and the HUI3 in Patients with Asthma. Value Health J. Int. Soc. Pharmacoeconomics Outcomes Res. **21**, 748–757 (2018). 10.1016/j.jval.2017.09.017 10.1016/j.jval.2017.09.017 PMC6026598 29909881

[29] Yang, F., Wong, C.K.H., Luo, N., Piercy, J., Moon, R., Jackson, J.: Mapping the kidney disease quality of life 36-item short form survey (KDQOL-36) to the EQ-5D-3L and the EQ-5D-5L in patients undergoing dialysis. Eur. J. Health Econ HEPAC Health Econ. Prev. Care. **20**, 1195–1206 (2019). 10.1007/s10198-019-01088-5 10.1007/s10198-019-01088-5 PMC6803593 31338698

[30] Kharroubi, S.A.: Analysis of SF-6D Health State Utility Scores: Is Beta Regression Appropriate? Healthc. Basel Switz. **8**, 525 (2020). 10.3390/healthcare8040525 10.3390/healthcare8040525 PMC7712516 33271844

[31] Hagiwara, Y., Shiroiwa, T., Taira, N., Kawahara, T., Konomura, K., Noto, S., Fukuda, T., Shimozuma, K.: Mapping EORTC QLQ-C30 and FACT-G onto EQ-5D-5L index for patients with cancer. Health Qual. Life Outcomes **18**, 354 (2020). 10.1186/s12955-020-01611-w 33143687 10.1186/s12955-020-01611-w PMC7641825

[32] Hunger, M., Eriksson, J., Regnier, S.A., Mori, K., Spertus, J.A., Cristino, J.: Mapping the kansas city cardiomyopathy questionnaire (KCCQ) Onto EQ-5D-3L in heart failure patients: results for the japanese and UK Value Sets. MDM Policy Pract. **5**, 2381468320971606 (2020). 10.1177/2381468320971606 33344768 10.1177/2381468320971606 PMC7727069

[33] Tsiachristas, A., Potter, C.M., Rocks, S., Peters, M., Cundell, M., McShane, R., Batchelder, L., Fox, D., Forder, J.E., Jones, K., Waite, F., Freeman, D., Fitzpatrick, R.: Estimating EQ-5D utilities based on the short-form long term conditions questionnaire (LTCQ-8). Health Qual. Life Outcomes **18**, 279 (2020). 10.1186/s12955-020-01506-w 32795317 10.1186/s12955-020-01506-w PMC7427949

[34] Ruzsa, G., Rencz, F., Brodszky, V.: Assessment of health state utilities in dermatology: an experimental time trade-off value set for the dermatology life quality index. Health Qual. Life Outcomes **20**, 87 (2022). 10.1186/s12955-022-01995-x 35658979 10.1186/s12955-022-01995-x PMC9164408

[35] Joyce, V.R., Sun, H., Barnett, P.G., Bansback, N., Griffin, S.C., Bayoumi, A.M., Anis, A.H., Sculpher, M., Cameron, W., Brown, S.T., Holodniy, M., Owens, D.K.: Mapping MOS-HIV to HUI3 and EQ-5D-3L in Patients With HIV. MDM Policy Pract. **2**, 2381468317716440 (2017). 10.1177/2381468317716440 30288427 10.1177/2381468317716440 PMC6125043

[36] Wailoo, A., Hernandez Alava, M., Escobar Martinez, A.: Modelling the relationship between the WOMAC Osteoarthritis Index and EQ-5D. Health Qual. Life Outcomes **12**, 37 (2014). 10.1186/1477-7525-12-37 24621311 10.1186/1477-7525-12-37 PMC4007823

[37] Gray, L.A., Hernandez Alava, M., Wailoo, A.J.: Mapping the EORTC QLQ-C30 to EQ-5D-3L in patients with breast cancer. BMC Cancer **21**, 1237 (2021). 10.1186/s12885-021-08964-5 34794404 10.1186/s12885-021-08964-5 PMC8600775

[38] Hernández Alava, M., Wailoo, A., Wolfe, F., Michaud, K.: The relationship between EQ-5D, HAQ and pain in patients with rheumatoid arthritis. Rheumatol. Oxf. Engl. **52**, 944–950 (2013). 10.1093/rheumatology/kes400 10.1093/rheumatology/kes400 PMC3630395 23339232

[39] Bilcke, J., Hens, N., Beutels, P.: Quality-of-life: a many-splendored thing? Belgian population norms and 34 potential determinants explored by beta regression. Qual. Life Res Int. J. Qual. Life Asp. Treat. Care Rehabil. **26**, 2011–2023 (2017). 10.1007/s11136-017-1556-y 10.1007/s11136-017-1556-y PMC5509833 28349241

[40] Villeneuve, C., Laroche, M.-L., Essig, M., Merville, P., Kamar, N., Coubret, A., Lacroix, I., Bouchet, S., Fruit, D., Marquet, P., Rousseau, A.: EPIGREN study group: Evolution and Determinants of Health-Related Quality-of-Life in Kidney Transplant Patients Over the First 3 Years After Transplantation. Transplantation **100**, 640–647 (2016). 10.1097/TP.0000000000000846 26569063 10.1097/TP.0000000000000846

[41] Esposito, P., Furini, F., Rampino, T., Gregorini, M., Petrucci, L., Klersy, C., Dal Canton, A., Dalla Toffola, E.: Assessment of physical performance and quality of life in kidney-transplanted patients: a cross-sectional study. Clin. Kidney J. **10**, 124–130 (2017). 10.1093/ckj/sfw102 28638612 10.1093/ckj/sfw102 PMC5469555

[42] Mouelhi, Y., Jouve, E., Alessandrini, M., Pedinielli, N., Moal, V., Meurette, A., Cassuto, E., Mourad, G., Durrbach, A., Dussol, B., Gentile, S.: Factors associated with Health-Related Quality of Life in Kidney Transplant Recipients in France. BMC Nephrol. **19**, 99 (2018). 10.1186/s12882-018-0893-6 29703170 10.1186/s12882-018-0893-6 PMC5921567

[43] Wang, Y., Hemmelder, M.H., Bos, W.J.W., Snoep, J.D., de Vries, A.P.J., Dekker, F.W., Meuleman, Y.: Mapping health-related quality of life after kidney transplantation by group comparisons: a systematic review. Nephrol. Dial. Transplant. Off. Publ. Eur. Dial Transpl. Assoc. - Eur. Ren. Assoc. **36**, 2327–2339 (2021). 10.1093/ndt/gfab232 10.1093/ndt/gfab232 PMC8643597 34338799

[44] Liem, Y.S., Bosch, J.L., Hunink, M.G.M.: Preference-based quality of life of patients on renal replacement therapy: a systematic review and meta-analysis. Value Health J. Int. Soc. Pharmacoeconomics Outcomes Res. **11**, 733–741 (2008). 10.1111/j.1524-4733.2007.00308.x 10.1111/j.1524-4733.2007.00308.x 18194399

[45] Wyld, M., Morton, R.L., Hayen, A., Howard, K., Webster, A.C.: A systematic review and meta-analysis of utility-based quality of life in chronic kidney disease treatments. PLoS Med. **9**, e1001307 (2012). 10.1371/journal.pmed.1001307 22984353 10.1371/journal.pmed.1001307 PMC3439392

[46] Glasziou, P.P., Cole, B.F., Gelber, R.D., Hilden, J., Simes, R.J.: Quality adjusted survival analysis with repeated quality of life measures. Stat. Med. **17**, 1215–1229 (1998). 10.1002/(sici)1097-0258(19980615)17:11%3c1215::aid-sim844%3e3.0.co;2-y 9670411 10.1002/(sici)1097-0258(19980615)17:11<1215::aid-sim844>3.0.co;2-y

[47] Herdman, M., Gudex, C., Lloyd, A., Janssen, M., Kind, P., Parkin, D., Bonsel, G., Badia, X.: Development and preliminary testing of the new five-level version of EQ-5D (EQ-5D-5L). Qual. Life Res Int. J. Qual. Life Asp. Treat. Care Rehabil. **20**, 1727–1736 (2011). 10.1007/s11136-011-9903-x 10.1007/s11136-011-9903-x PMC3220807 21479777

[48] Andrade, L.F., Ludwig, K., Goni, J.M.R., Oppe, M., de Pouvourville, G.: A French Value Set for the EQ-5D-5L. Pharmacoeconomics **38**, 413–425 (2020). 10.1007/s40273-019-00876-4 31912325 10.1007/s40273-019-00876-4 PMC7080328

[49] Hernandez Alava, M., Sasso, A., Hnynn Si, P.E., Gittus, M., Powell, R., Dunn, L., Thokala, P., Fotheringham, J.: Relationship between standardized measures of chronic kidney disease-associated pruritus intensity and health-related quality of life measured with the EQ-5D Questionnaire: A Mapping Study. Acta Derm. Venereol. **103**, adv11604 (2023). 10.2340/actadv.v103.11604 37731210 10.2340/actadv.v103.11604 PMC10522326

