# Supplementary material for: Predicting EQ-5D-3L utility values from clinical data in a prospective cohort of kidney transplant recipients
Source: Eur J Health Econ. 2025 Jun 11;27(1):17–28. doi: 10.1007/s10198-025-01802-6 (PMC12929319; doi:10.1007/s10198-025-01802-6)
Supplement: Supplementary file 1 — Supplementary file1 (PDF 157 KB) [file 10198_2025_1802_MOESM1_ESM.pdf]

Predicting EQ-5D-3L utility values from clinical data in a prospective cohort of kidney transplant recipients

V. Bonnemains, Y. Foucher, P. Tessier, C. David, M. Giral, E. Dantan; for the DIVAT Consortium*

Journal: The European Journal of Health Economics

Corresponding author: Etienne Dantan, Nantes Université, Univ Tours, INSERM, MethodS in Patients-centered outcomes and HEalth Research, SPHERE, F-44000 Nantes, France. IRS2, 22 boulevard Bénoni Goullin, 44200 Nantes, France. Phone: +33 2 53 00 91 28, Email: Etienne.Dantan@univ-nantes.fr

DIVAT (Données Informatisées et VAlidées en Transplantation) Consortium:

Lyon E. Hériot : Lionel Badet, Maria Brunet, Fanny Buron, Rémi Cahen, Ricardo Codas, Sameh Daoud, Valérie Dubois, Coralie Fournie, François Gaillard, Arnaud Grégoire, Alice Koenig, Charlène Lévi, Emmanuel Morelon, Claire Pouteil-Noble, Maud Rabeyrin, Thomas Rimmelé, Olivier Thaunat ; **Montpellier :** Nicolas Abdo, Sylvie Delmas, Moglie Le Quintrec, Vincent Pernin, Hélène Perrochia, Jean-Emmanuel Serre, Ilan Szwarc ; **Nancy :** Alice Aarnink, Asma Alla, Pascal Eschwege, Luc Frimat, Sophie Girerd, Jacques Hubert, Raphaël Kormann, Marc Ladriere, François Lagrange, Emmanuelle Laurain, Pierre Lecoanet, Jean-Louis Lemelle ; Anthony Mannuguerra, Charles Mazeaud, Michael Peres ; **Nantes :** Gilles Blancho, Julien Branchereau, Diego Cantarovich, Agnès Chapelet, Jacques Dantal, Clément Deltombe, Lucile Figueres, Raphael Gaisne, Claire Garandeau, Magali Giral, Caroline Gourraud-Vercel, Maryvonne Hourmant, Georges Karam, Clarisse Kerleau, Delphine Kervella, Christophe Masset, Aurélie Meurette, Simon Ville, Christine Kandell, Anne Moreau, Karine Renaudin, Florent Delbos, Alexandre Walencik, Anne Devis ; **Nice :** Laetitia Albano, Damien Ambrosetti,

Nadia Ben Hassen, Mathilde Blois, Marion Cremoni, Matthieu Durand, Patricia Goldis, Clément Gosset, Fatimaezzahra Karimi, Antoine Sicard, Giorgo Toni **; Paris-Necker :** Lucile Amrouche, Dany Anglicheau, Olivier Aubert, Lynda Bererhi, Christophe Legendre, Alexandre Loupy, Frank Martinez, Arnaud Méjean, Rébecca Sberro-Soussan, Anne Scemla, Marc-Olivier Timsit, Julien Zuber ; **Paris-Saint-Louis :** Gillian Divard, Carmen Lefaucheur ; **Saint-Etienne :** Christophe Mariat, Guillaume Claisse.

Details of each modeling approach

Linear Mixed Model:

The linear mixed model assumes a linear link between health-state utility values (HSUV) and patients' characteristics. The latter can have a fixed effect β (the corresponding covariates are noted X in the following equations) and/or a random patient-specific effect b (corresponding covariates are noted Z in the following equations). The residuals and the random effects are assumed to follow centered normal distributions and to be independent and identically distributed. Letting σ_ϵ denote the standard deviation of the residuals and Σ_b denote the covariance matrix of the random effects, we can model the HSUV u of the ith patient's jth observation as follows:

$$\begin{cases} u_{ij} = X_{ij}^T\beta + Z_{ij}^T b_i + \epsilon_{ij} \\ \epsilon_{ij} \sim \mathcal{N}(0, \sigma_\epsilon) \\ b_i \sim \mathcal{N}(0, \Sigma_b) \end{cases}$$

Adjusted Limited Dependent Variable Mixture Model (ALDVMM):

The ALDVMM also assumes a linear link between HSUVs and patients' characteristics but considers the existence of several latent classes c in the population, i.e. unobserved groups of patients on which the covariates do not have the same effect. Hence, the fixed effects β become class-dependent β_c. Moreover, the ALDVMM consider the lowest possible HSUV ψ_1 and the next-to highest possible HSUV ψ_2:

$$u_{ijc}^* = X_{ij}^T\beta_c + Z_{ij}^T b_i + \epsilon_{ij}$$

$$u_{ijc} = \begin{cases} \psi_1 \; si \; u_{ijc}^* \leq \psi_1 \\ u_{ijc}^* \; si \; u_{ijc}^* \in]\psi_1; \psi_2] \\ 1 \; si \; u_{ijc}^* > \psi_2 \end{cases}$$

The class membership probability can be modelled with a logit link. Letting δ denote the effects of covariates W on this probability:

$$P(c|W_i) = \frac{e^{\delta_c W_i}}{\sum_s e^{\delta_s W_i}}$$

Finally, we can predict patient i's jth observed HSUVs as follows:

$$E\left(u_{ij}\middle|X,Z,W\right) = \sum_c P(c|W) \times E(u_{ijc}|X,Z)$$

Beta regression:

The beta distribution is defined on the]0;1[interval with two shape parameters μ and φ, Γ denoting the gamma distribution:

$$\mathrm{f}(\mathrm{y}|\mu,\phi) = \frac{\Gamma(\phi)}{\Gamma(\mu\phi)\Gamma\left((1-\mu)\phi\right)} \times y^{\mu\phi-1} \times (1-\mathrm{y})^{(1-\mu)\phi-1}$$

One can see φ as a dispersion parameter, while the expected value of y is equal to μ. The beta regression consists in modeling this expected value using a link function (typically logit) as follows:

$$\ln\left(\frac{\mu_{ij}}{1-\mu_{ij}}\right) = X_{ij}^T\beta + Z_{ij}^T b_i$$

Two-part beta regression:

In the presence of a zero-inflated distribution, one can complete this model by including a second sub-model in order to predict the probability π of observing a y value of 0 given the fixed effect δ of covariates W and the random effect d of covariates U:

$$\begin{cases} \ln\left(\frac{\mu_{ij}}{1-\mu_{ij}}\right) = X_{ij}^T\beta + Z_{ij}^T b_i \\ \ln\left(\frac{\pi_{ij}}{1-\pi_{ij}}\right) = W_{ij}^T\delta + U_{ij}^T d_i \end{cases}$$

Patient i's jth observation's expected value can then be computed as follows:

$$E\left(y_{ij}\middle|X,Z,U,W\right) = P\left(y_{ij} \neq 0\right) \times E\left(y_{ij}\middle|X,Z,y_{ij} \neq 0\right)$$

Table S1. Multivariate beta mixed model of patients' rescaled decrement in health-state utility values (HSUVs) (n=2774, 13 patients excluded due to missing data on adjustment covariates). A greater decrement indicates a lower HSUV.

	Coef.	95% CI	p
**Baseline predictors**			
Age (years)	0.011	[0.008, 0.015]	<0.001
Sex (men vs. women)	-0.470	[-0.567, -0.373]	<0.001
Body mass index (> 30 kg/m^2, vs ≤ 30)	0.223	[0.080, 0.366]	0.002
Initial nephropathy (relapsing vs. not relapsing)	-0.118	[-0.224, -0.012]	0.029
Recipient history of diabetes (yes vs. no)	0.390	[0.248, 0.533]	<0.001
Recipient history of cardiovascular disease (yes vs. no)	0.238	[0.134, 0.341]	<0.001
Time spent on dialysis (years)	0.044	[0.030, 0.058]	<0.001
**Slope of the evolution (years)**			
≤ 1-year post-KT	-0.356	[-0.499, -0.214]	<0.001
> 1-year post-KT	0.033	[0.024, 0.043]	<0.001

Intercept: -2.761 (95% CI [-3.000, -2.522]), SD of the random intercept: 1.014 (95% CI [0.895, 1.150]), SD of the random slope in the first year post-KT: 0.764 (95% CI [0.571, 1.023]).

CI, confidence interval; KT, kidney transplantation; SD, standard deviation

Table S2. Mixed multivariate ALDVMM of patient's health-state utility values (n=2,775, 12 patients excluded due to missing data on adjustment covariates).

CLASS MEMBERSHIP	**Coef.**	**95% CI**	**p**
**Baseline predictors**			
Intercept	0.604	[0.447, 0.761]	<0.001
CLASS 1	**Coef.**	**95% CI**	**p**
**Baseline predictors**			
Age (years)	-0.001	[-0.0013, -0.0004]	0.001
Sex (men vs. women)	0.043	[0.029, 0.058]	<0.001
Recipient history of cardiovascular disease (yes vs. no)	-0.025	[-0.039, -0.010]	0.001
**Slope of the evolution (years)**			
≤ 1-year post-KT	0.066	[0.040, 0.092]	<0.001
> 1-year post-KT	-0.002	[-0.003, -0.0002]	0.022
Intercept: 0.894 (95% CI [0.860, 0.929]), SD of the random intercept: 0.170, SD of the random slope in the first year post-KT: 0.176, SD of the residuals: 0.065 (95% CI [0.058, 0.071]).			
CLASS 2	**Coef.**	**95% CI**	**p**
**Baseline predictors**			
Age (years)	-0.003	[-0.004, -0.002]	<0.001
Sex (men vs. women)	0.124	[0.088, 0.159]	<0.001
Recipient history of diabetes (yes vs. no)	-0.134	[-0.179, -0.089]	<0.001
Recipient history of cardiovascular disease (yes vs. no)	-0.032	[-0.070, 0.005]	0.089
Time spent on dialysis (years)	-0.009	[-0.013, -0.005]	<0.001
**Slope of the evolution (years)**			
> 1-year post-KT	-0.011	[-0.015, -0.007]	<0.001
Intercept: 0.817 (95% CI [0.738, 0.896]), SD of the random intercept: 0.170, SD of the random slope in the first year post-KT: 0.176, SD of the residuals: 0.264 (95% CI [0.251, 0.277]).			

ALDVMM, Adjusted limited dependent variable mixture model; CI, confidence interval; KT, kidney transplantation; SD, standard deviation
