# Supplementary material for: Predicting EQ-5D-3L utility values from clinical data in a prospective cohort of kidney transplant recipients
Source: Eur J Health Econ. 2025 Jun 11;27(1):17–28. doi: 10.1007/s10198-025-01802-6 (PMC12929319; doi:10.1007/s10198-025-01802-6)
Supplement: Supplementary file 2 — Supplementary file2 (PDF 379 KB) [file 10198_2025_1802_MOESM2_ESM.pdf]

Predicting EQ-5D-3L utility values from clinical data in a prospective cohort of kidney transplant recipients

V. Bonnemains, Y. Foucher, P. Tessier, C. David, M. Giral, E. Dantan; for the DIVAT Consortium*

Journal: The European Journal of Health Economics

Corresponding author: Etienne Dantan, Nantes Université, Univ Tours, INSERM, MethodS in Patients-centered outcomes and HEalth Research, SPHERE, F-44000 Nantes, France. IRS2, 22 boulevard Bénoni Goullin, 44200 Nantes, France. Phone: +33 2 53 00 91 28, Email: Etienne.Dantan@univ-nantes.fr

DIVAT (Données Informatisées et VAlidées en Transplantation) Consortium:

Lyon E. Hériot : Lionel Badet, Maria Brunet, Fanny Buron, Rémi Cahen, Ricardo Codas, Sameh Daoud, Valérie Dubois, Coralie Fournie, François Gaillard, Arnaud Grégoire, Alice Koenig, Charlène Lévi, Emmanuel Morelon, Claire Pouteil-Noble, Maud Rabeyrin, Thomas Rimmelé, Olivier Thaunat ; **Montpellier :** Nicolas Abdo, Sylvie Delmas, Moglie Le Quintrec, Vincent Pernin, Hélène Perrochia, Jean-Emmanuel Serre, Ilan Szwarc ; **Nancy :** Alice Aarnink, Asma Alla, Pascal Eschwege, Luc Frimat, Sophie Girerd, Jacques Hubert, Raphaël Kormann, Marc Ladriere, François Lagrange, Emmanuelle Laurain, Pierre Lecoanet, Jean-Louis Lemelle ; Anthony Mannuguerra, Charles Mazeaud, Michael Peres ; **Nantes :** Gilles Blancho, Julien Branchereau, Diego Cantarovich, Agnès Chapelet, Jacques Dantal, Clément Deltombe, Lucile Figueres, Raphael Gaisne, Claire Garandeau, Magali Giral, Caroline Gourraud-Vercel, Maryvonne Hourmant, Georges Karam, Clarisse Kerleau, Delphine Kervella, Christophe Masset, Aurélie Meurette, Simon Ville, Christine Kandell, Anne Moreau, Karine Renaudin, Florent Delbos, Alexandre Walencik, Anne Devis ; **Nice :** Laetitia Albano, Damien Ambrosetti,

Nadia Ben Hassen, Mathilde Blois, Marion Cremoni, Matthieu Durand, Patricia Goldis, Clément Gosset, Fatimaezzahra Karimi, Antoine Sicard, Giorgo Toni **; Paris-Necker :** Lucile Amrouche, Dany Anglicheau, Olivier Aubert, Lynda Bererhi, Christophe Legendre, Alexandre Loupy, Frank Martinez, Arnaud Méjean, Rébecca Sberro-Soussan, Anne Scemla, Marc-Olivier Timsit, Julien Zuber ; **Paris-Saint-Louis :** Gillian Divard, Carmen Lefaucheur ; **Saint-Etienne :** Christophe Mariat, Guillaume Claisse.

Sensitivity analysis

First-time KT recipients

Out of the 2,787 included patients, 2,378 (85%) underwent KT for the first time. These patients completed 4,868 questionnaires, which corresponds to an average of 2.0 questionnaires per patient.

Fitting the prediction models on this subpopulation results in similar predictions as the one obtained for the whole population. The models' estimated coefficients when exclusively including first-time KT recipients are very close to the ones obtained when including the whole population (Figure S1). As a result, the models' performances in terms of calibration and precision do not differ from the original models' (Figure S2, Table S1).

Tables

Table S1. Root mean squared error (RMSE), mean absolute error (MAE) and prediction range for each model when exclusively including first-time kidney-transplant recipients (n = 2,378).

	RMSE	MAE	Prediction range
Linear mixed model	0.222	0.163	[0.507, 0.980]
Beta mixed model	0.227	0.152	[0.507, 0.952]
Two-part beta mixed model	0.192	0.126	[0.427, 0.990]
2-class mixed ALDVMM	0.224	0.159	[0.642, 0.937]
Model proposed by Li et al.[*]	0.229	0.168	[0.718, 0.883]

[*]Model 7 from Li et al.'s Table 3, including recipients' age, diabetic status and gender as predictors.

Figures

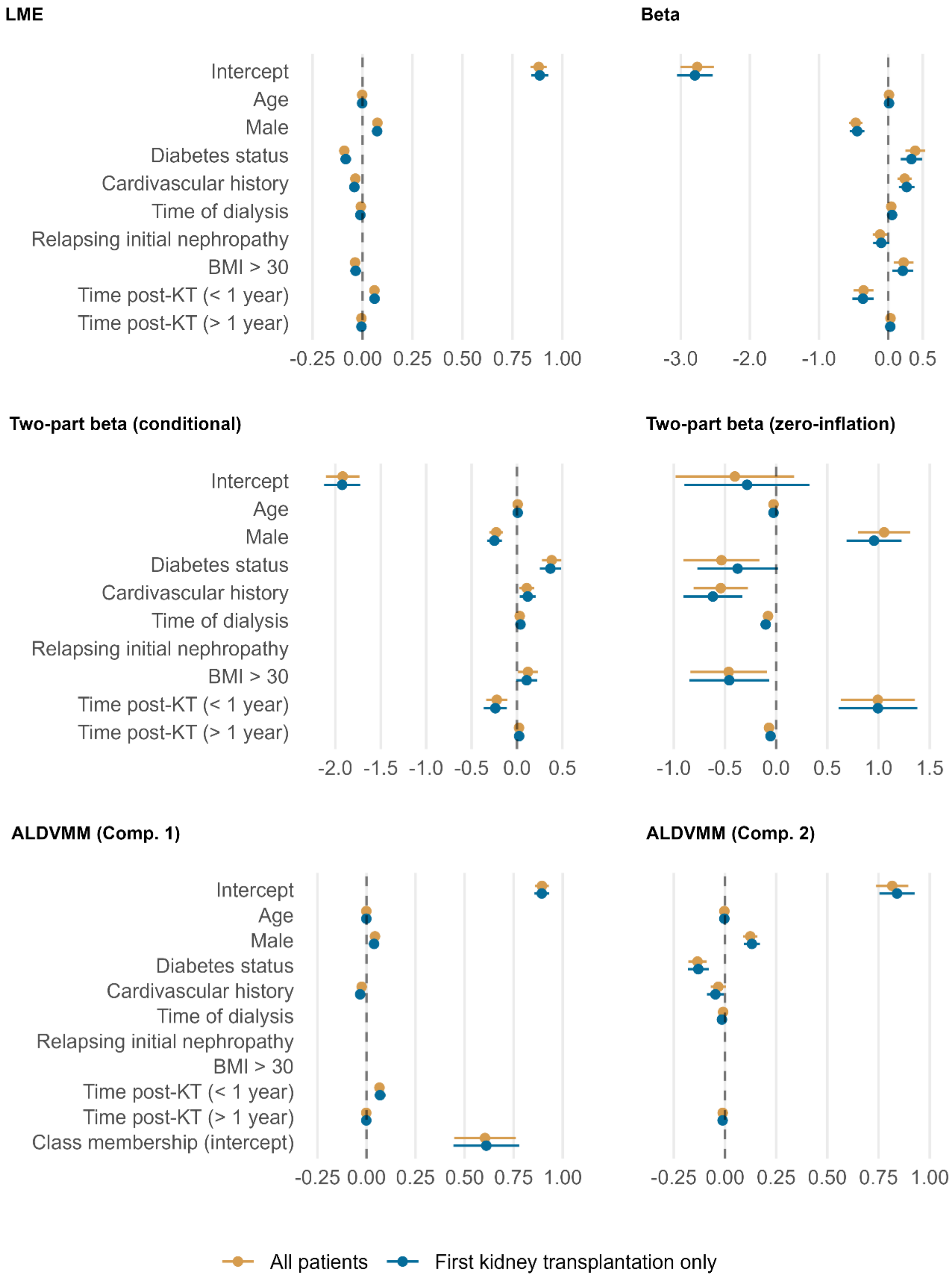

<u>Figure S1.</u> Comparison of the regression coefficients for first-kidney-transplants (n = 2,378, blue) versus all transplantations (n = 2,787, yellow)

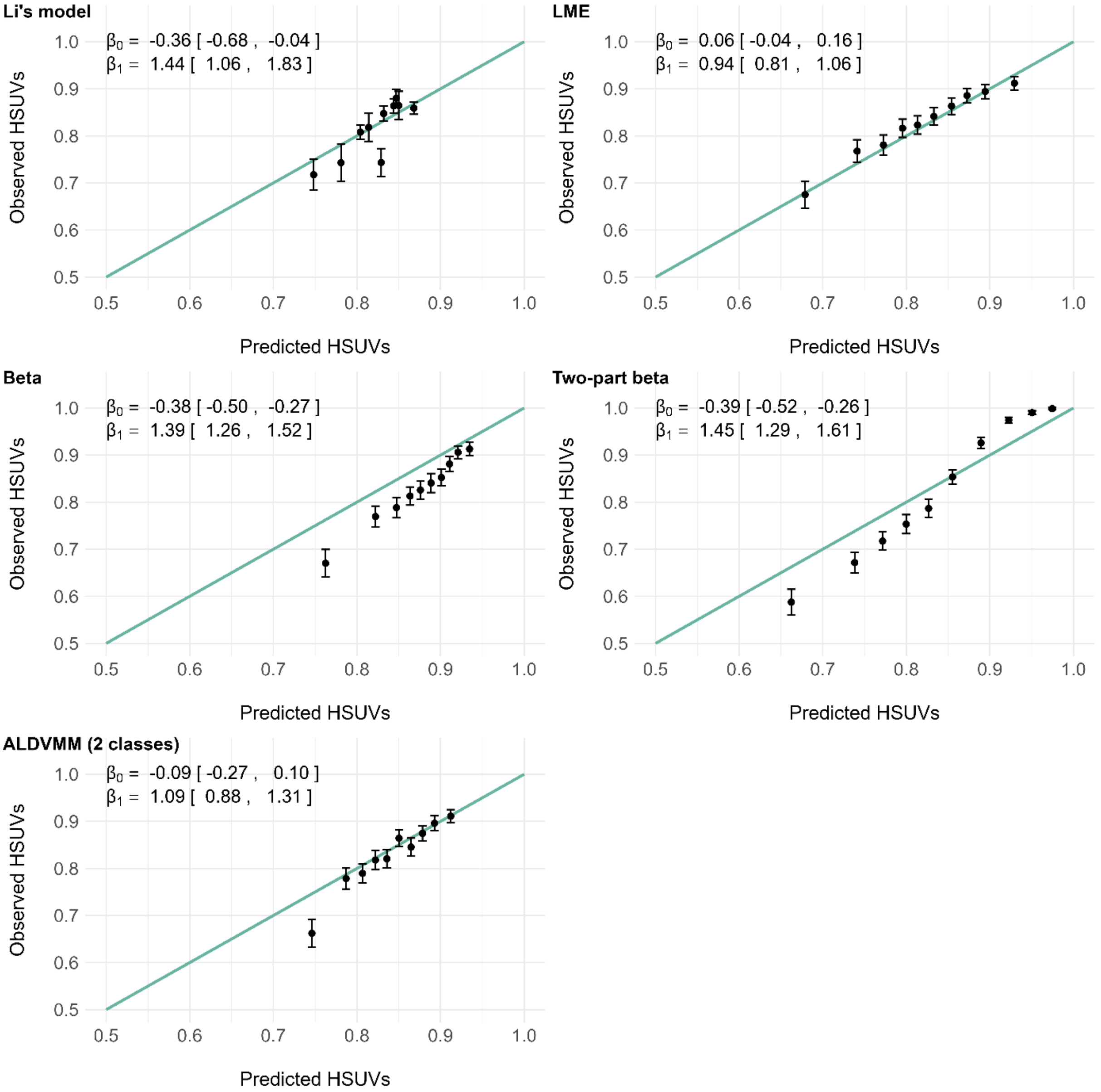

Figure S2. Predicted versus observed health-state utility values (HSUVs) for the five models considering only first-kidney transplants (n=2,378). The predicted HSUVs were grouped by deciles, and the black dots represent the means of the observed and predicted HSUVs for each group. The green lines represent perfect calibrations. β_0 and β_1 are the intercept and the slope, respectively, obtained by linearly regressing* the observed HSUVs and the ten predicted deciles (a perfect calibration corresponding to β_0 = 0 and β_1 =1). LME: linear mixed effect model; ALDVMM: adjusted limited dependent variable mixture model.

* Li's model's 5th decile and the 2-class ALDVMM's 1st decile were detected as outliers by analysing studentised residuals and Cook's distance and excluded for β_0 and β_1 estimation.
