# Supplementary material for: Predicting EQ-5D-3L utility values from clinical data in a prospective cohort of kidney transplant recipients
Source: Eur J Health Econ. 2025 Jun 11;27(1):17–28. doi: 10.1007/s10198-025-01802-6 (PMC12929319; doi:10.1007/s10198-025-01802-6)
Supplement: Supplementary file 3 — Supplementary file3 (PDF 561 KB) [file 10198_2025_1802_MOESM3_ESM.pdf]

Predicting EQ-5D-3L utility values from clinical data in a prospective cohort of kidney transplant recipients

V. Bonnemains, Y. Foucher, P. Tessier, C. David, M. Giral, E. Dantan; for the DIVAT Consortium*

Journal: The European Journal of Health Economics

Corresponding author: Etienne Dantan, Nantes Université, Univ Tours, INSERM, MethodS in Patients-centered outcomes and HEalth Research, SPHERE, F-44000 Nantes, France. IRS2, 22 boulevard Bénoni Goullin, 44200 Nantes, France. Phone: +33 2 53 00 91 28, Email: Etienne.Dantan@univ-nantes.fr

DIVAT (Données Informatisées et VAlidées en Transplantation) Consortium:

Lyon E. Hériot : Lionel Badet, Maria Brunet, Fanny Buron, Rémi Cahen, Ricardo Codas, Sameh Daoud, Valérie Dubois, Coralie Fournie, François Gaillard, Arnaud Grégoire, Alice Koenig, Charlène Lévi, Emmanuel Morelon, Claire Pouteil-Noble, Maud Rabeyrin, Thomas Rimmelé, Olivier Thaunat ; **Montpellier :** Nicolas Abdo, Sylvie Delmas, Moglie Le Quintrec, Vincent Pernin, Hélène Perrochia, Jean-Emmanuel Serre, Ilan Szwarc ; **Nancy :** Alice Aarnink, Asma Alla, Pascal Eschwege, Luc Frimat, Sophie Girerd, Jacques Hubert, Raphaël Kormann, Marc Ladriere, François Lagrange, Emmanuelle Laurain, Pierre Lecoanet, Jean-Louis Lemelle ; Anthony Mannuguerra, Charles Mazeaud, Michael Peres ; **Nantes :** Gilles Blancho, Julien Branchereau, Diego Cantarovich, Agnès Chapelet, Jacques Dantal, Clément Deltombe, Lucile Figueres, Raphael Gaisne, Claire Garandeau, Magali Giral, Caroline Gourraud-Vercel, Maryvonne Hourmant, Georges Karam, Clarisse Kerleau, Delphine Kervella, Christophe Masset, Aurélie Meurette, Simon Ville, Christine Kandell, Anne Moreau, Karine Renaudin, Florent Delbos, Alexandre Walencik, Anne Devis ; **Nice :** Laetitia Albano, Damien Ambrosetti,

Nadia Ben Hassen, Mathilde Blois, Marion Cremoni, Matthieu Durand, Patricia Goldis, Clément Gosset, Fatimaezzahra Karimi, Antoine Sicard, Giorgo Toni **; Paris-Necker :** Lucile Amrouche, Dany Anglicheau, Olivier Aubert, Lynda Bererhi, Christophe Legendre, Alexandre Loupy, Frank Martinez, Arnaud Méjean, Rébecca Sberro-Soussan, Anne Scemla, Marc-Olivier Timsit, Julien Zuber ; **Paris-Saint-Louis :** Gillian Divard, Carmen Lefaucheur ; **Saint-Etienne :** Christophe Mariat, Guillaume Claisse.

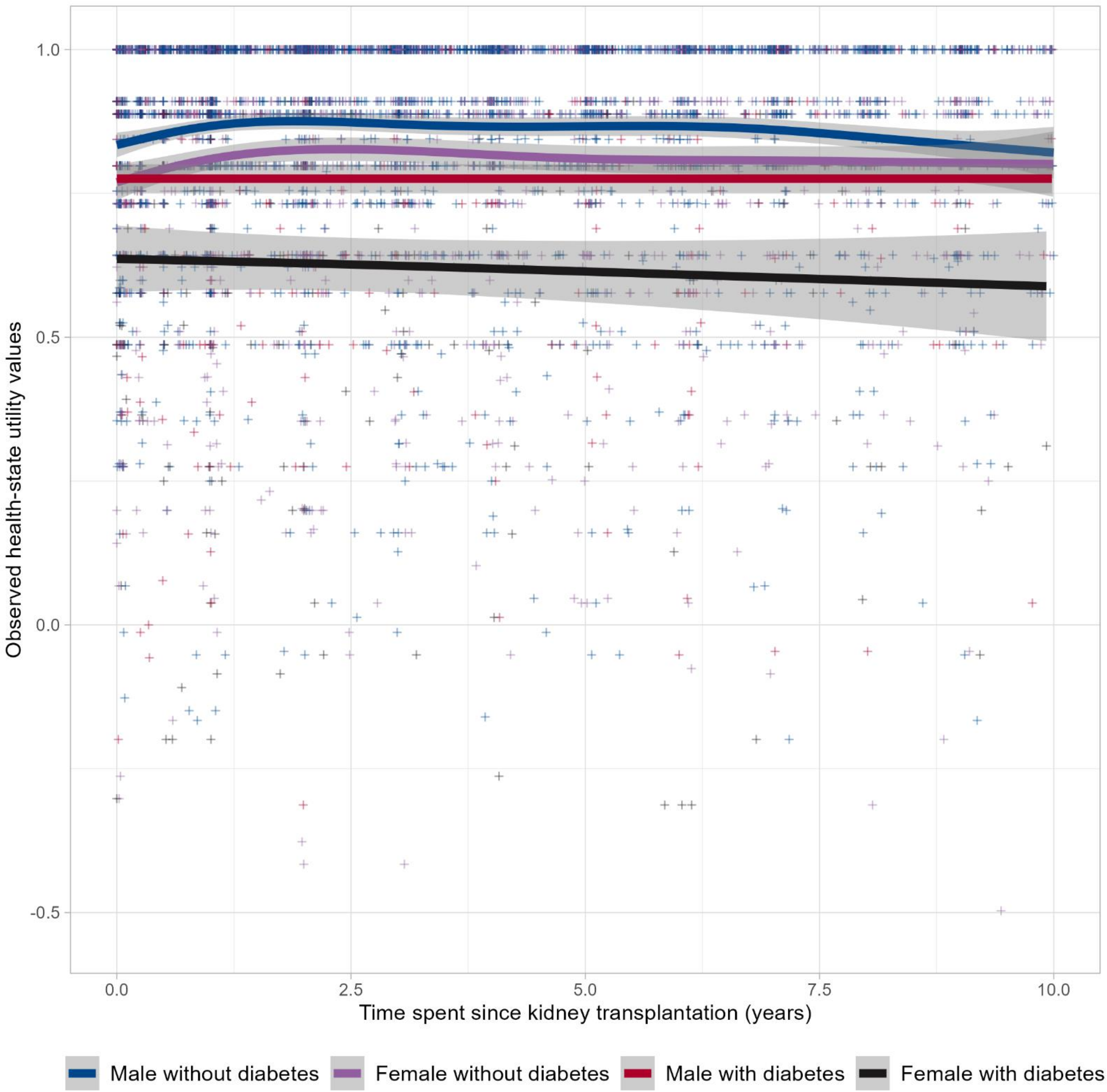

Figure S1. Observed health-state utility values (HSUVs) according to recipients' sex and diabetic status. The points represent the observations, whereas the lines represent a smoothed average estimation using a generalised additive modelling with the grey areas representing 95% confidence intervals [2].

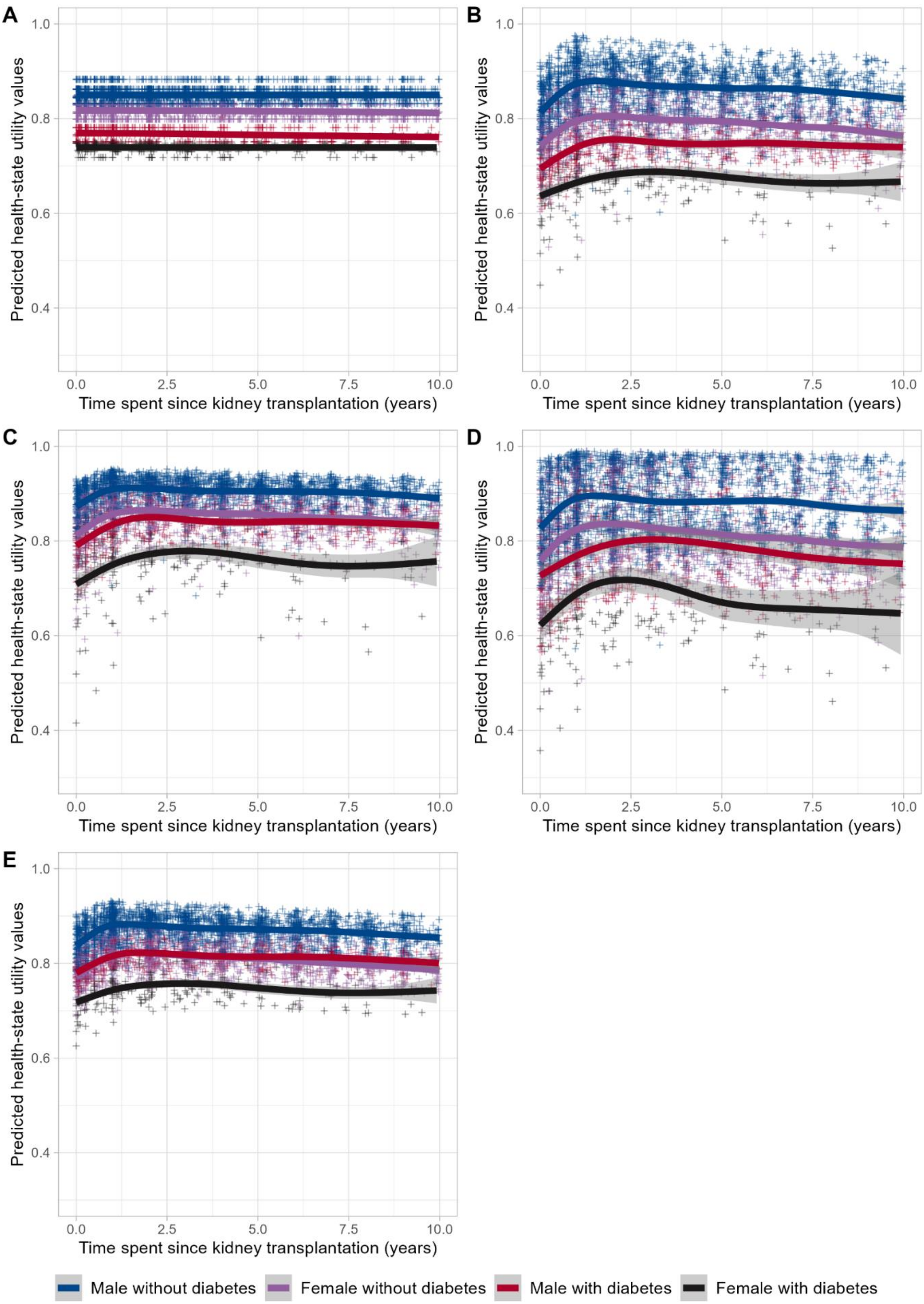

Figure S2. Health-state utility values (HSUVs) predicted using **A-**the model proposed by Li et al.[1]; **B-** the linear mixed model; **C-**the beta mixed model; **D-**the two-part beta mixed model; and **E-**the mixed ALDVMM, according to recipients' sex and diabetic status. The points represent the model's predictions, whereas the lines represent a smoothed average estimation using a generalised additive modelling with the grey areas representing 95% confidence intervals [2].

References

1. Li, B., Cairns, J.A., Draper, H., Dudley, C., Forsythe, J.L., Johnson, R.J., Metcalfe, W., Oniscu, G.C., Ravanan, R., Robb, M.L., Roderick, P., Tomson, C.R., Watson, C.J.E., Bradley, J.A.: Estimating Health-State Utility Values in Kidney Transplant Recipients and Waiting-List Patients Using the EQ-5D-5L. Value Health J. Int. Soc. Pharmacoeconomics Outcomes Res. 20, 976–984 (2017). https://doi.org/10.1016/j.jval.2017.01.011
2. Wood, S.N.: Generalized Additive Models: An Introduction with R, Second Edition. Chapman and Hall/CRC, New York (2017)
